# Wastewater-Based Surveillance of SARS-CoV-2 and Modeling of COVID-19 Infection Trends

**DOI:** 10.3390/tropicalmed10090264

**Published:** 2025-09-16

**Authors:** Wenli Wang, Ruoyu Li, Shilin Chen, Liangping Chen, Yu Jiang, Jianjun Xiang, Jing Wu, Jing Li, Zhiwei Chen, Chuancheng Wu

**Affiliations:** 1 School of Public Health, Fujian Medical University, Fuzhou 350122, China; wangwenli@fjmu.edu.cn (W.W.); 18313150887@163.com (R.L.); 18876266031@163.com (S.C.); clp43210@163.com (L.C.); jiangyu@fjmu.edu.cn (Y.J.); jianjun.xiang@fjmu.edu.cn (J.X.); jingwu@fjmu.edu.cn (J.W.); leejing@fjmu.edu.cn (J.L.); 2 Key Laboratory of Environment and Health, Fujian Province University, Fuzhou 350122, China; 3 The Affiliated Fuzhou Center for Disease Control and Prevention of Fujian Medical University, Fuzhou 350000, China

**Keywords:** SARS-CoV-2, COVID-19, viral load, time-series analysis, wastewater-based epidemiology, early warning, public health monitoring

## Abstract

Background: This study was performed to evaluate the early warning value of wastewater-based epidemiology (WBE) in monitoring severe acute respiratory syndrome coronavirus 2 (SARS-CoV-2) and its correlation with population-level coronavirus disease 2019 (COVID-19) infection trends. Methods: Wastewater samples from Fuzhou’s Sewage Treatment Plant A were concentrated via membrane filtration and quantified using reverse transcription quantitative polymerase chain reaction (RT-qPCR). Viral load data were integrated with sentinel hospital positivity rates and respiratory outpatient visits from 11 city hospitals. Stratified cross-correlation lag analysis was performed by gender, age, and hospital type. Results: Using the lowest single-day genome concentration as a proxy for daily SARS-CoV-2 levels was advantageous. Wastewater viral concentrations correlated positively with clinical cases, with peaks preceding reports by 0 to 17 days. Stratified analysis further indicated that women, older adults, and individuals from general hospitals were more sensitive to changes in wastewater viral loads, showing stronger correlations between infection trends and wastewater signals. Conclusions: Wastewater surveillance of SARS-CoV-2 can effectively predict COVID-19 infection trends and offers a scientific basis for stratified and targeted interventions. The findings underscore the value of WBE as an early warning tool in public health surveillance.

## 1. Introduction

Since the first identification of severe acute respiratory syndrome coronavirus 2 (SARS-CoV-2) in humans in late 2019, the world has experienced multiple waves of coronavirus disease 2019 (COVID-19) outbreaks. To date, the virus has caused nearly 780 million confirmed cases and over 7 million deaths worldwide [1]. The rapid transmission of the virus, along with the continual emergence of new variants, particularly the Omicron variant and its sub-lineages, has significantly increased the complexity of pandemic control efforts [2].

Although respiratory droplets and direct contact are considered the primary routes of SARS-CoV-2 transmission [3], numerous studies have shown that the virus can survive in the digestive tract of infected individuals and be shed in feces [4]. While there is currently no definitive clinical evidence confirming fecal–oral transmission [5], the consistent detection of viral RNA in feces and wastewater has drawn increasing attention. In particular, the presence of high concentrations of viral RNA in community wastewater suggests its potential as a valuable indicator for public health surveillance. Notably, although the infectious activity of SARS-CoV-2 in the environment declines rapidly, its RNA can remain relatively stable in wastewater and be detectable for several days [6,7]. This persistence of viral RNA signals highlights its potential as an early warning indicator for localized outbreaks.

In recent years, wastewater-based epidemiology (WBE) has emerged as a novel population health surveillance tool and has been widely applied for COVID-19 monitoring in North America, Europe, and Australia. Studies in these regions have demonstrated that WBE can often detect an upward trend in infections earlier than clinical reporting systems [8,9]. In China, pilot wastewater surveillance programs have been implemented in cities such as Beijing, Shanghai, and Hong Kong [10]. However, most of these studies have focused on viral RNA detection or viral profiling during peak epidemic periods. Systematic research that integrates long-term clinical surveillance data with predictive model development remains relatively limited. In particular, there are no publicly reported studies in Fujian Province or Fuzhou City that conduct continuous WBE monitoring combined with lag effect analysis.

Compared with traditional epidemic surveillance methods that rely on clinically confirmed case reports, WBE offers key advantages such as being noninvasive and covering a broad population base, making it particularly suitable in settings with limited clinical testing resources or a high proportion of asymptomatic infections. WBE signals can change several days to weeks before an increase in reported case numbers, giving it strong potential for early warning [11]. Moreover, WBE has been successfully applied to monitor various viruses, including norovirus, poliovirus, and adenovirus, in public health contexts [12]. The COVID-19 pandemic has further accelerated global interest in and research on WBE, establishing it as an important supplementary tool for forecasting epidemic trends, guiding public health interventions, and informing resource allocation.

This study focused on Fuzhou City as the study area, utilizing reverse transcription quantitative polymerase chain reaction (RT-qPCR) to quantify SARS-CoV-2 viral loads in wastewater. By integrating sentinel hospital COVID-19 positivity rates and outpatient data for respiratory illnesses from 11 hospitals, we conducted time-series and lag effect analyses to explore the relationship between viral trends in wastewater and community infection dynamics. The findings aim to provide scientific support for developing an environmental surveillance-based regional early warning system and enhancing the public health response to emerging infectious diseases.

## 2. Materials and Methods

### 2.1. Collection of Wastewater Samples and Viral Detection

Wastewater samples in this study were collected from the influent of the A Wastewater Treatment Plant in Fuzhou City. The plant serves the Jiangbei central urban area, an important part of Fuzhou’s built-up city, covering approximately 76 square kilometers and a population of about 1.5 million, which accounts for 23.76% of the city’s total urban population (6.314 million). This area has a high population density and considerable mobility, meaning that residents frequently move within the city for daily activities such as commuting, shopping, and social interactions. As a mixed-use district that integrates residential, commercial, and workplace functions, most daily life and wastewater generation occur within the plant’s service area, making it a representative location for wastewater-based epidemiological monitoring.

Sampling was conducted every three days from 1 January to 31 December 2023. The procedure followed the Method for enrichment and nucleic acid detection of SARS-CoV-2 in sewage (WS/T 799-2022) [13]. Collect one 24 h mixed sample at the inlet of the sewage treatment plant every three days, and transport and store it under refrigeration conditions. Complete virus concentration and RNA extraction within 24 h.

**Figure 1:**
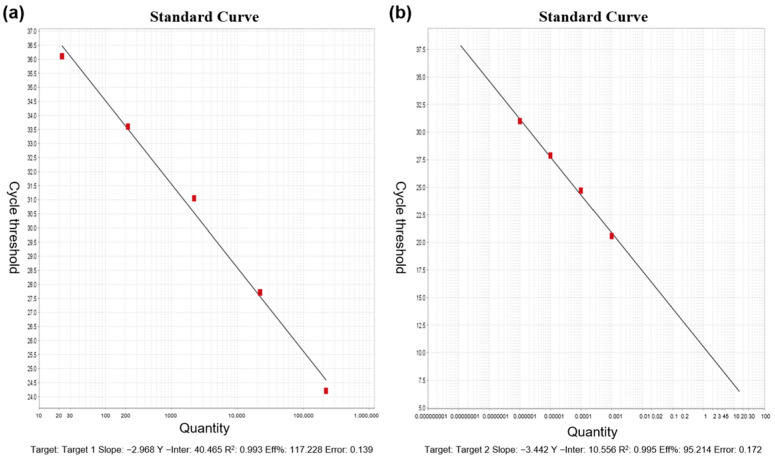
Absolute quantification standard curves for SARS-CoV-2 N1 and N2 genes: (**a**) N1 gene; (**b**) N2 gene.

**Figure 2:**
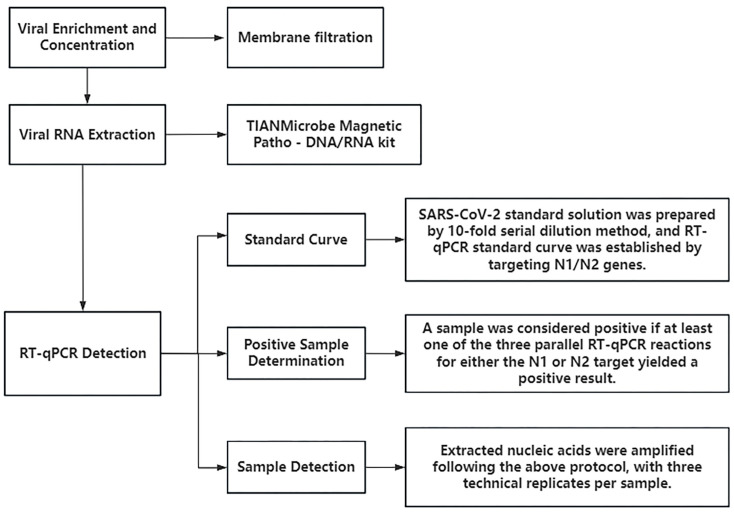
Sample processing and testing workflow.

Detection of viral RNA was performed using RT-qPCR, targeting the N1 and N2 regions of the SARS-CoV-2 nucleocapsid (N) gene. These targets have been shown to offer high sensitivity [14], with studies reporting greater sensitivity for *N* gene detection compared to the ORF1 region [15]. The absolute quantification standard curves for the SARS-CoV-2 N1 and N2 genes are shown in Figure 1, with a minimum detection limit (MDL) of 2.21 gene copies/µL and a maximum detection limit of 2.21 × 10^5^ gene copies/µL. The detailed sample processing and detection workflow is depicted in Figure 2.

### 2.2. Sources of COVID-19 Surveillance and Respiratory Outpatient Data

Following the Protocol for Prevention and Control of COVID-19 (10th Edition), this study was conducted within the framework of the National Influenza Surveillance Network. Sentinel influenza hospitals are designated hospitals within this network. In this study, two national influenza sentinel hospitals in Fuzhou were selected to conduct SARS-CoV-2 surveillance among outpatients with influenza-like illness (ILI), inpatients with severe acute respiratory infections (SARIs), and other suspected COVID-19 cases. As part of the surveillance system, these sentinel hospitals routinely provide clinical care, testing, and data collection for relevant cases. Patients included in the surveillance were either those who sought care directly at the sentinel hospitals or those referred from other institutions; severe cases presenting at these hospitals were also included. During the surveillance process, approximately 10% of cases were randomly selected each day for SARS-CoV-2 testing. All monitoring data were provided by the laboratory system of the Fuzhou Center for Disease Control and Prevention, and specimens were collected exclusively from the two sentinel hospitals.

In addition, respiratory outpatient visit records were collected from 11 hospitals in Fuzhou, including 10 general hospitals and 1 children’s hospital. Based on the The International Statistical Classification of Diseases and Related Health Problems 10th Revision (ICD-10), included cases were those coded J00–J99, covering a range of respiratory diseases. The outpatient data contained no personally identifiable information.

### 2.3. Data Processing and Analysis

#### 2.3.1. Basic Data Collection

This study analyzed the relationship between SARS-CoV-2 detection in wastewater and COVID-19 infection trends in Fuzhou, China. Viral loads were measured using RT-qPCR, targeting the N1 and N2 genes. CT values were converted to absolute concentrations based on standard curves. Hospital infection data were processed as follows: from January to December 2023, the daily SARS-CoV-2 positivity rate was obtained from two sentinel hospitals in Fuzhou. The positivity rate refers to the proportion of patients with respiratory illnesses who tested positive for SARS-CoV-2 on a given day, among those who were tested, rather than the positivity rate in the general population. Although this does not represent the entire population, it remains the most accessible and objective indicator of community exposure currently available. To estimate the daily number of infections in Fuzhou, this positivity rate was multiplied by the total number of daily outpatient visits for respiratory diseases (ICD-10 codes J00–J99) across 11 hospitals in the city. This variable was treated as an absolute number and was not converted into an infection rate; therefore, no general population denominator was applied. The purpose of this approach was to combine a relatively stable positivity rate with the patient visit volume to create a proxy indicator of daily infection levels, which was then used for time-series and lag correlation analyses with wastewater viral loads.

#### 2.3.2. Data Preprocessing

A comprehensive database was constructed by integrating SARS-CoV-2 viral load data from wastewater, positivity rates from sentinel hospitals, and respiratory outpatient case counts. Data cleaning procedures were then performed to remove duplicate records, logical inconsistencies, and missing information to ensure data quality. For improved comparability and analytical stability, wastewater viral load data were uniformly converted to virus concentrations expressed in log_10_ (log_10_ VL).

#### 2.3.3. Statistical Analysis Methods

Descriptive statistical methods were used to examine monthly and daily trends in viral load and positivity rates, alongside estimations of daily infection counts based on respiratory outpatient data. To verify the match between lag time and infection trends, lag analysis was introduced, building on existing research methods [16,17,18]. The correlation between viral load and the number of infections at different lag times was evaluated using R 4.4.2 software. The optimal lag period was determined by taking the magnitude of the correlation coefficient as the goodness-of-fit index, thereby revealing the potential of wastewater viral signals to prospectively predict clinical infection trends. Meanwhile, Python’s Matplotlib library (version 3.9.3) was used to visualize the changes in viral load and infection trends, providing an intuitive representation of their dynamic relationship.

To further quantify the association between SARS-CoV-2 levels and COVID-19 infections, a generalized linear model (GLM) was applied [19]. Specifically, a Poisson-based GLM was employed with adjustments for overdispersion and autocorrelation in the data, using time-series data constructed from date, day of the week, and health outcome indicators (daily outpatient visits). The final set of covariates included in the model was determined by minimizing the quasi-Akaike information criterion (Q-AIC) [20,21]. The GLM was specified as follows:

log*E*(*Y_t_*) = *βZ_t_* + ns(*time*, 6 × 1) + *DOW* + intercept

where *E*(*Y_t_*) represents the expected number of COVID-19 cases on day *t*; *β* is the exposure-response coefficient; *Z_t_* denotes the SARS-CoV-2 concentration in wastewater on day t; ns() is the natural spline smoothing function with specified degrees of freedom; *time* is the date variable; *DOW* represents dummy variables for the day-of-week effect; and intercept is the model constant.

## 3. Results

### 3.1. Overall Detection of SARS-CoV-2

As shown in Table 1, a total of 132 wastewater samples were collected and analyzed in 2023. The detection rate for the SARS-CoV-2 N1 gene was 69.70%, while that for the N2 gene was 67.42%. When considering a sample positive if either the N1 or N2 gene was detected (N1∪N2; the union can improve the overall sensitivity), the overall positivity rate increased to 84.09%. Conversely, if both genes were required to be detected simultaneously (N1∩N2; the intersection can improve the overall specificity), the positivity rate dropped to 53.03%. Further analysis revealed that the combined detection rate (N1∪N2) remained consistently high throughout the year, with the lowest monthly detection rate still reaching 53.85%. In contrast, the concurrent detection rate (N1∩N2) showed greater variability, peaking at 100% in June but falling below 20% in March and November. Given the stability and representativeness of the combined detection approach, all subsequent analyses in this study used the N1∪N2 criterion to determine SARS-CoV-2 positivity, ensuring the reliability and consistency of the results. The stability and representativeness of the joint detection method can more comprehensively capture signals of virus presence, reduce information loss caused by limitations in detection methods, and more robustly reflect the actual epidemic status of the virus in the population. Therefore, all subsequent analyses of this study used the (N1∪N2) standard to determine the positivity of novel coronavirus, to ensure the reliability and consistency of the results.

### 3.2. Detection of SARS-CoV-2 Viral Load

Based on absolute quantification using standard curves, the SARS-CoV-2 viral load in concentrated wastewater samples collected from Fuzhou’s A Wastewater Treatment Plant in 2023 ranged from 4.83 to 9.96 × 10^8^ gene copies/µL for the N1 gene, and from 2.59 to 3.32 × 10^9^ gene copies/µL for the N2 gene (see Table 2). Since the N1 and N2 gene regions do not allow for differentiation among SARS-CoV-2 variants, and relying on a single gene target may not accurately reflect the overall viral burden, this study selected the lower concentration between N1 and N2 on each sampling day (denoted as N3) to represent the minimum viral load, and the higher concentration (denoted as N4) as the maximum viral load. This dual-indicator approach offers a more comprehensive assessment of the dynamic fluctuations in SARS-CoV-2 levels in wastewater. The analysis revealed distinct viral load peaks in January, June, and October.

### 3.3. COVID-19 Testing Results from Sentinel Hospitals

From January to December 2023, the monthly SARS-CoV-2 positivity rates reported by sentinel hospitals in Fuzhou are presented in Table 3. The results show that the positivity rate in January was significantly higher than in other months, likely due to the relaxation of public health restrictions following policy adjustments at the end of 2022, which may have led to a short-term surge in infections. In the subsequent months, the positivity rate remained at relatively low levels, indicating a transition into a period of lower viral circulation. However, a noticeable rebound was observed in May, suggesting a brief resurgence of infections. This uptick may be attributed to waning population immunity or the emergence of new viral variants. As time progressed, the positivity rate gradually declined, reflecting a stabilization in the epidemic trend.

### 3.4. Comparison of SARS-CoV-2 Viral Load Trends and Estimated Infection Trends Based on Sentinel Hospital Positivity Rates

**Figure 3:**
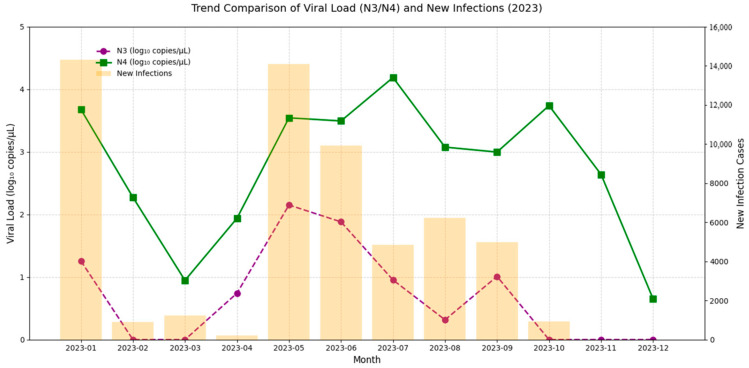
Comparative trend analysis of SARS-CoV-2 viral load in wastewater and estimated COVID-19 cases in Fuzhou, China, in 2023.

**Figure 4:**
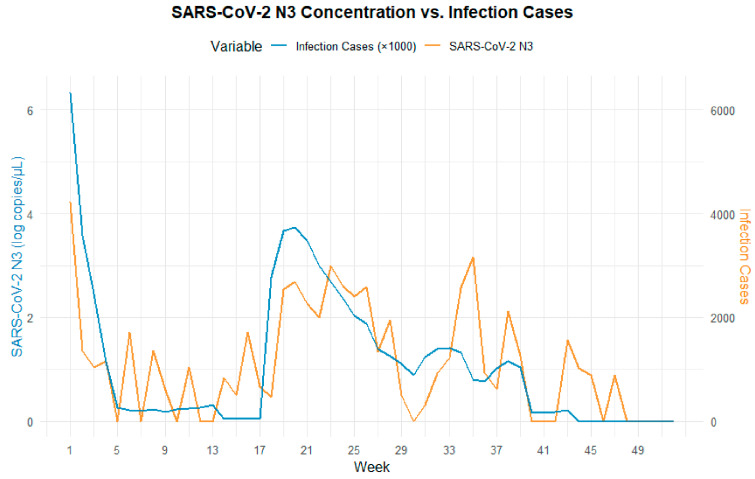
Comparative trend analysis of low viral load per single wastewater test (N3) and estimated COVID-19 cases.

Figure 3 illustrates the comparison between the SARS-CoV-2 viral load in wastewater (log_10_ VL) and the estimated number of infections derived from sentinel hospital positivity rates in Fuzhou during 2023. The results indicate that the N3 trend more closely mirrors changes in estimated infection numbers. To further examine the relationship between N3 and infection trends, weekly summaries of viral load and infection estimates were analyzed (Figure 4). The findings show a strong correlation between wastewater viral load and infection case numbers. In contrast, N4 displayed irregular spikes in certain months, which may have obscured the true trend of infections and resulted in a weaker correlation with case numbers. Therefore, N3 was selected as the representative variable for subsequent cross-correlation and lag effect analysis.

### 3.5. Lag Analysis Between SARS-CoV-2 Viral Load in Wastewater and Infection Cases

#### 3.5.1. Lag Analysis Between Viral Load and Total Estimated Infections

**Figure 5:**
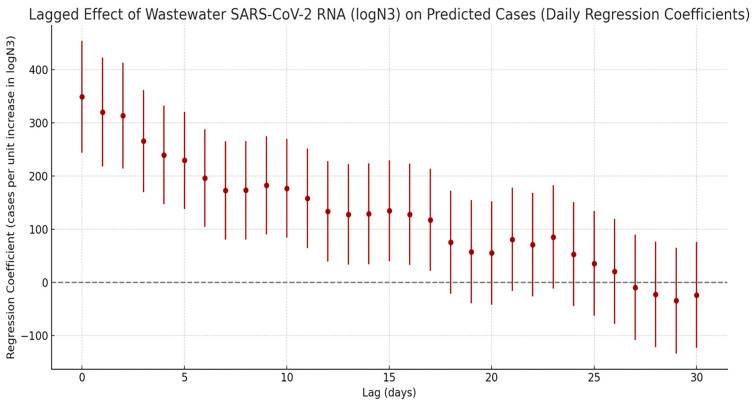
Lagged effects of SARS-CoV-2 exposure on the number of COVID-19 infections.

A cross-correlation lag effect analysis was performed between SARS-CoV-2 N3 concentrations and estimated infection numbers derived from sentinel hospital positivity rates (Figure 5). The results revealed a significant temporal lag relationship between viral load in wastewater and infection cases. The regression coefficient peaked at lag 0 days, indicating a strong positive correlation and suggesting that increases in wastewater viral concentration are most closely aligned with the surge in clinical cases on the same day, reflecting a sharp rise in short-term infection risk. Beyond this point, the correlation gradually declined with increasing lag time. Within 10 days, the regression coefficients remained within the range of 100–200. By day 18, the lower bound of the confidence interval crossed zero, indicating that the association was no longer statistically significant. These findings suggest that the effective early warning window for wastewater surveillance is approximately 0–17 days, meaning that peaks in viral load can precede clinical case reports by up to 17 days, offering a valuable lead time for public health interventions.

#### 3.5.2. Lag Analysis Between Viral Load and Gender-Specific Infection Cases

**Figure 6:**
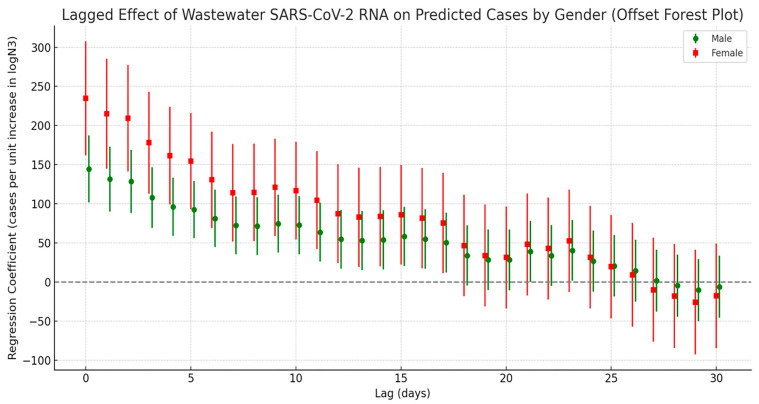
Lag effect of SARS-CoV-2 exposure on the number of COVID-19 cases by gender.

Stratified cross-correlation analysis (Figure 6) showed that SARS-CoV-2 N3 concentrations in wastewater were most strongly positively correlated with both male and female infection counts at a lag of 0 days. The strength of the correlation gradually declined over time and became statistically insignificant by the third week, suggesting that the predictive value of wastewater signals is primarily concentrated within the first two weeks following viral exposure. While the overall correlation trends between N3 and infection numbers were similar for both sexes, notable differences were observed between genders. Stratified results indicated that females have a stronger association with viral load: at lag 0 days, the regression coefficient for females was 234.9, which was markedly higher than that for males (144.7). This disparity remained consistently significant throughout the 0–17-day lag period. In contrast, the male group exhibited only sporadic secondary peaks at lags of 21 and 23 days, potentially reflecting delayed response effects or behavioral differences.

#### 3.5.3. Lag Analysis Between Viral Load and Age-Specific Infection Cases

**Figure 7:**
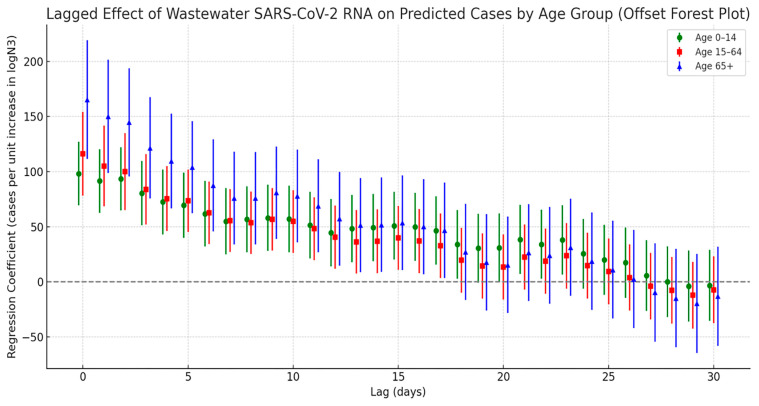
Lag effect of SARS-CoV-2 exposure on COVID-19 case numbers across different age groups.

Figure 7 presents the cross-correlation lag analysis between SARS-CoV-2 viral load in wastewater and infection cases across different age groups. Overall, all age groups showed the strongest correlation with viral load at lag day 0, with the strength of the correlation gradually decreasing as the lag time increased. However, the correlation patterns varied notably across age groups. Among the elderly population aged 65 and above, the regression coefficient peaked at lag day 0 and gradually declined over the following days, remaining significant up to day 17. In the 0–14 age group (children and adolescents), a significant positive correlation was also observed on lag day 0 (peak value = 98.2), with a secondary significant peak appearing at lags of 21 to 23 days. In contrast, the 15–64 age group (adults) exhibited intermediate correlation values between the other two groups, with relatively moderate fluctuations throughout the lag period.

### 3.6. Wastewater SARS-CoV-2 Viral Load and Lag Associations with Outpatient Visits

#### 3.6.1. Lag Analysis Between Viral Load and Outpatient Visits by Hospital Type

**Figure 8:**
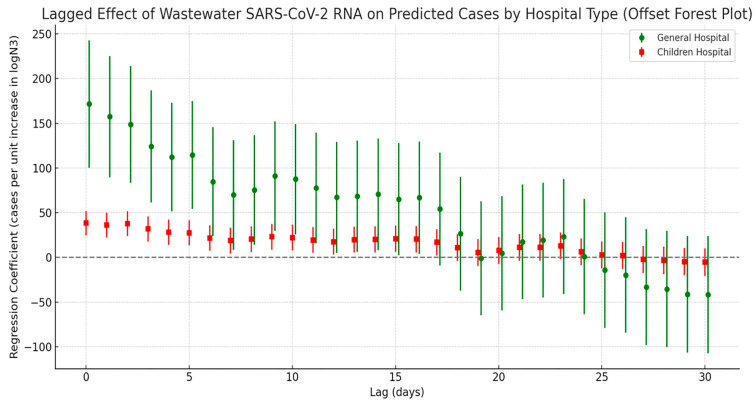
Lag effect of SARS-CoV-2 exposure on COVID-19 case numbers across different hospitals.

The lagged correlation analysis between wastewater SARS-CoV-2 N3 concentrations and estimated COVID-19 infections from different hospital types (general hospitals and the children’s hospital) revealed a pattern of initially strong and then weakening correlations over time (see Figure 8). Specifically, general hospitals exhibited the highest regression coefficient at lag day 0, indicating that wastewater viral signals can serve as an immediate early warning indicator for patient surges in general hospitals. In contrast, the regression coefficients for the children’s hospital were comparatively lower; however, the significance of the lag effect persisted over a longer duration. This suggests that the time window between viral exposure and clinical presentation may be longer in pediatric populations, possibly due to differences in disease progression or transmission dynamics among children.

#### 3.6.2. Lag Effect Between Viral Load and Outpatient Visits by Sex

**Figure 9:**
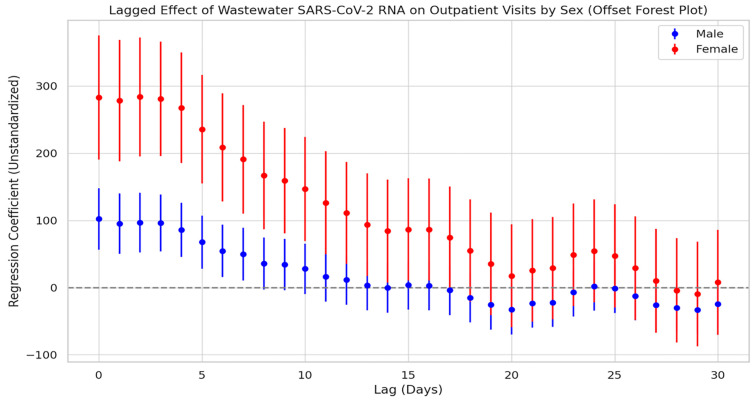
Lag effect of SARS-CoV-2 exposure on respiratory outpatient visits by gender.

Figure 9 presents the lagged regression analysis between wastewater SARS-CoV-2 N3 concentrations and respiratory outpatient visits among males and females. Overall, both groups showed a rapid rise in correlation during the short lag period (approximately 0–3 days), peaking around lag day 3. This was followed by a gradual decline in correlation strength with increasing lag time, becoming statistically insignificant after 2 to 3 weeks. These results suggest that the predictive window of wastewater viral load for outpatient trends is relatively limited in duration. Notably, while both male and female groups peaked at a similar lag time, the regression coefficients for females consistently exceeded those for males throughout the lag period, with the difference remaining statistically significant. This indicates a stronger and more sustained association between viral load and outpatient visits among females.

#### 3.6.3. Lag Effect Between Viral Load and Outpatient Visits by Age Group

**Figure 10:**
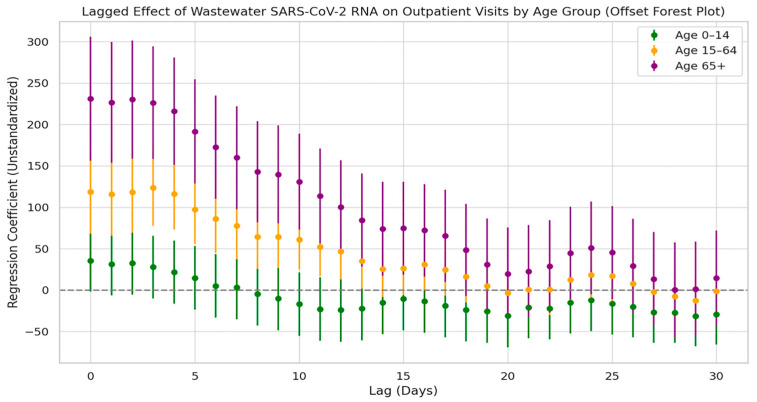
Lag effect of SARS-CoV-2 exposure on respiratory outpatient visits by age groups.

Figure 10 illustrates the lagged regression relationship between SARS-CoV-2 N3 concentrations in wastewater and respiratory outpatient visits across different age groups. The results show that all three age groups exhibited strong positive correlations during the early lag period: the elderly group reached its peak regression coefficient on lag day 0, the adult group peaked between lag days 0 and 3, and the children and adolescents group also peaked around lag day 0. After these peak points, the regression coefficients gradually declined with increasing lag time.

Among the three groups, the elderly population showed the highest regression coefficients and the longest duration of statistically significant lag effects—lasting up to 17 days—indicating the strongest temporal alignment with wastewater viral concentrations. The adult group had intermediate coefficients with a relatively stable trend and no secondary peaks. The children and adolescents group showed the lowest coefficients, and the 95% confidence intervals crossed the baseline at multiple lag days, suggesting no statistically significant lag effect. These findings indicate that elderly individuals are more sensitive to environmental exposure to the virus.

## 4. Discussion

This study, based on 2023 data from Fuzhou, China, examined the association between SARS-CoV-2 concentrations in wastewater and COVID-19 infection trends. Wastewater samples were analyzed using RT-qPCR with multiple targets (N1, N2, N3, and N4), and infection numbers were estimated from sentinel hospital positivity rates to conduct cross-correlation lag analyses assessing the predictive potential of wastewater surveillance for epidemic dynamics. While such integrated data approaches have been applied in international WBE studies [22,23,24], our research offers several unique contributions: (1) a direct comparison of multiple target concentrations to assess their sensitivity to case trends, identifying the N3 daily minimum concentration as most strongly synchronized with case counts; (2) stratified trend analyses by population subgroups (older adults, females, and patients visiting general hospitals), enabling fine-scale monitoring of potential risks in vulnerable populations; and (3) coverage of a large portion of the municipal wastewater network, capturing both spatial and temporal features of cryptic community transmission. Our findings indicate that SARS-CoV-2 concentrations in wastewater are significantly and positively correlated with outpatient case counts, with peaks often preceding reported cases by 0–17 days. These results not only support our hypothesis that wastewater surveillance can reflect hidden community transmission but also provide new empirical evidence for the application of WBE in localized interventions, vulnerable population risk assessment, and multi-target optimization strategies.

Detection using a single target gene is susceptible to environmental interference and RNA degradation, increasing the risk of false positives or false negatives and potentially reducing the accuracy of SARS-CoV-2 detection in wastewater [25]. To improve reliability, the World Health Organization recommended in its 2021 wastewater surveillance guidelines the use of two or more conserved gene targets for combined detection [26]. In this study, we adopted a combined detection criterion using both N1 and N2 gene targets (N1∪N2) to determine positivity. Results showed that this approach yielded a significantly higher detection rate compared to simultaneous positivity for both genes (N1∩N2), with lower variability, indicating that N1∪N2 offer greater sensitivity and representativeness. A similar strategy has been widely implemented by the U.S. Centers for Disease Control and Prevention in its national SARS-CoV-2 wastewater surveillance program to better capture viral signals during periods of low community transmission [27]. A study in Italy also confirmed that combining *ORF1ab* and *N* gene targets improved detection sensitivity by 34%, especially in low-viral-load scenarios [28]. Furthermore, our findings revealed notable fluctuations in the N1∩N2 detection rate, which may be attributed to meteorological factors. For example, Fuzhou experienced higher rainfall in March and November, potentially diluting viral RNA in sewage and reducing the likelihood of simultaneous detection of both gene targets [29,30]. Conversely, the elevated detection rate observed in June may be linked to increased temperatures, which could accelerate viral shedding [31]. Therefore, to enhance monitoring efficacy, wastewater surveillance protocols should incorporate meteorological data. During rainy seasons, prioritizing the N1∪N2 criterion may help maintain sensitivity, while during dry periods, the stricter N1∩N2 standard could be used to improve specificity.

Building upon the combined gene target detection approach, this study further introduced the use of N3 as a representative indicator of SARS-CoV-2 viral load in wastewater. This strategy aims to mitigate the potential distortion of trend interpretation caused by anomalously elevated concentrations in a single gene target. The rationale behind this design lies in the observation that, within multi-target detection schemes, the lowest value often better reflects the true viral concentration [32]. Our results demonstrated that N3 exhibited significantly greater consistency with trends in estimated infection cases compared to N4, suggesting that N3 offers superior representativeness and explanatory power when assessing the intensity of community transmission. This finding aligns with existing research indicating that lower concentration values are less likely to be inflated by environmental factors and may more accurately reflect the baseline level of viral spread in the community [33]. For instance, the abnormal peak in N4 observed in January 2023 may have been driven by a short-term surge in infections following the relaxation of COVID-19 control measures at the end of 2022. In contrast, N3 more closely tracked the actual decline in transmission following the Spring Festival period.

The lag effect between viral load in wastewater and reported infection cases (ranging from 0 to 17 days) is one of the core findings of this study. It not only validates the practical potential of WBE for outbreak forecasting but also aligns well with results from international studies. Mechanistically, the concentration of viral RNA in wastewater reflects the aggregate shedding from the entire community, including a substantial proportion of undiagnosed or asymptomatic individuals. In contrast, clinical case reporting involves a natural delay due to the progression from infection to symptom onset, healthcare seeking, and eventual diagnosis [34,35]. In this study, the viral load indicator (N3) showed the strongest regression relationship with estimated infection numbers at a lag of 0 days, suggesting a high degree of synchronicity between wastewater signals and clinical data in densely populated urban settings. This may be attributed to faster healthcare access and robust medical infrastructure in cities [36,37,38]. The observed maximum lag window of 17 days provides a valuable early-response period for public health interventions. For instance, during the early stages of an unusual rise in viral load, actions such as targeted community testing and environmental disinfection can be initiated, offering a more proactive approach compared to traditional surveillance systems reliant solely on clinical reports. This lag range is highly consistent with findings from several international studies. For example, research conducted in the United States, Canada, and Pakistan has commonly shown that wastewater viral concentrations can predict changes in case numbers 0 to 14 days in advance [15,35,39,40,41]. It should be noted that although the 0–17-day time window provides a considerable buffer for public health responses, its wide range may introduce uncertainty in resource allocation during actual interventions. Factors such as population density, socioeconomic status, and differences in public health infrastructure can all influence the interval between viral signals and the subsequent rise in cases [42,43,44]. For example, in areas with high population density or limited public health resources, increases in cases may appear more rapidly. In contrast, in low-density areas or regions with poor healthcare accessibility, clinical reporting may be delayed. Future studies could conduct stratified analyses of these factors to narrow the uncertainty in lag-based predictions, thereby enhancing the operational utility of early warnings.

Additionally, this study examined the lag effects between wastewater viral load and infection trends across different demographic groups, revealing notable disparities in both response timing and correlation strength. Female infection counts consistently exhibited higher regression coefficients with viral concentration compared to males, which may be attributed to greater health awareness and more proactive healthcare-seeking behaviors among women. A population-based survey in China reported that females had significantly higher rates of healthcare utilization during the pandemic and were more likely to report mild symptoms promptly [45]. Similarly, a cross-sectional study in Saudi Arabia found that women were more inclined to report mild symptoms during COVID-19 outbreaks [46], while research from Germany observed that women were more likely to engage in protective behaviors [47]. This heightened sensitivity to health risks may further strengthen the temporal alignment between infection trends and wastewater viral signals in the female population. Age-stratified analysis showed that individuals aged 65 and older demonstrated the strongest and most sustained correlation with wastewater viral loads. This is likely due to a combination of factors, including shorter incubation periods, faster disease progression, and lower healthcare-seeking delays in older adults [48]. In contrast, children aged 0–14 exhibited a secondary peak in correlation at 21–23 days, suggesting a longer incubation period or delayed care-seeking behavior in this group [49]. Supporting this, a multinational cross-sectional study found that 18.6% of caregivers of children reported delaying emergency care due to fears of COVID-19 exposure [50], potentially extending the lag between wastewater signals and clinical case reporting in pediatric populations.

Analysis by hospital type further reinforced population-specific differences: outpatient infection counts at general hospitals showed a peak correlation with viral concentration at a 0-day lag, indicating strong immediacy, whereas correlations at children’s hospitals were weaker but more prolonged. This pattern likely reflects diagnostic delays or atypical symptom presentation among pediatric patients [51,52,53]. These findings underscore the need for public health resource allocation strategies to account for behavioral and healthcare utilization differences across population subgroups. Additionally, analysis of respiratory outpatient visit data revealed that the predictive window of the wastewater viral load on clinic utilization was primarily concentrated within a 0–3-day lag, after which the association rapidly declined. Among all age groups, the elderly exhibited the strongest and longest-lasting response to viral signals. These results highlight the potential of WBE for early warning applications, especially in dynamically monitoring high-risk populations and informing targeted resource prioritization.

This study further validates the application value of WBE in the routine prevention and control of COVID-19, particularly in contexts marked by delayed case reporting and high proportions of asymptomatic infections, where WBE can offer more timely risk signals [54]. By utilizing N3 as a key monitoring indicator and applying a 0–17-day lag window, it is possible to develop a closed-loop system linking wastewater surveillance, risk warning, and public health response. This would enable early identification and targeted intervention during outbreaks. For populations shown to be more sensitive to changes in viral load, such as women and the elderly, increasing sampling frequency is recommended. In contrast, for children who exhibit delayed responses, extending the early warning period and enhancing wastewater surveillance at key locations may be more effective. The multi-target detection strategy and lag analysis approach used in this study demonstrate strong generalizability and could be expanded to monitor other respiratory pathogens such as influenza and respiratory syncytial virus (RSV) [55,56]. Notably, countries such as the United States, the Netherlands, and Turkey have already incorporated WBE into their national surveillance systems [57,58], offering valuable reference models for the development of an independent and efficient wastewater monitoring and early warning platform in China.

Although this study provides strong evidence for the application of WBE in dynamic monitoring and risk warning of COVID-19, it has certain limitations. First, the concentration of viral RNA in wastewater is susceptible to environmental factors such as temperature, pH, and rainfall, yet meteorological variables were not systematically incorporated to control for potential confounding effects. Second, the sampling interval was every three days, which may not fully capture rapid fluctuations in viral concentrations during periods of accelerated transmission or short-term variability. This frequency was determined as a trade-off between temporal resolution and resource availability; in future studies, particularly during epidemic peaks, daily sampling could be considered to more accurately reflect the dynamics of viral load. In addition, while the RT-qPCR method used here targets both N1 and N2 genes to improve sensitivity, it cannot resolve viral subtypes. In the context of frequent strain replacement, this limitation may underestimate differences in transmission risk among variants. Future research could integrate metagenomic sequencing to achieve the dual goals of strain typing and epidemic monitoring. Furthermore, the estimation of infection numbers in this study was based on positivity rates from sentinel hospitals, which may be biased by differences in healthcare-seeking behaviors among demographic groups. To improve predictive accuracy, subsequent work could incorporate community-based surveys and integrate data on vaccination coverage, population mobility, and meteorological factors to develop a multi-source, integrated early warning model. Furthermore, the scope of WBE could be expanded to the joint monitoring of other pathogens, thereby promoting the intelligent and routine development of environmental health surveillance systems.

## 5. Conclusions

This study demonstrates that monitoring the SARS-CoV-2 viral load in wastewater effectively reflects community-level COVID-19 infection trends and offers an early warning window of 0–17 days. The observed differences in correlation strength across population subgroups suggest that wastewater-based epidemiology can provide valuable insights for early intervention among high-risk groups. Building on stratified analysis, this study highlights the potential of wastewater surveillance in multidimensional population health management and rapid outbreak response. Future efforts may expand toward variant tracking, spatiotemporal modeling, and multi-pathogen early warning systems to enhance dynamic monitoring and precision control of infectious disease transmission.

## Figures and Tables

**Table 1:** Overall detection of SARS-CoV-2 in wastewater in Fuzhou, China, in 2023.

Month	Number of Tests (N1/N2)	Positive Rate (%)			
		N1	N2	N1∪N2	N1∩N2
January	12	75.00	83.33	100.00	58.33
February	10	70.00	50.00	80.00	40.00
March	11	18.18	63.64	63.64	18.18
April	12	83.33	75.00	83.33	75.00
May	11	100.00	90.91	100.00	90.91
June	8	100.00	100.00	100.00	100.00
July	11	90.91	63.64	100.00	54.55
August	12	75.00	58.33	83.33	50.00
September	10	90.00	90.00	100.00	80.00
October	11	45.45	63.64	72.73	36.36
November	11	63.64	54.55	81.82	36.36
December	13	38.46	30.77	53.85	15.38
Total	132	69.70	67.42	84.09	53.03

**Table 2:** SARS-CoV-2 viral load at wastewater treatment plant A in Fuzhou, China, in 2023.

Month	Ct Value		Viral Load (Gene Copies/µL)				Log_10_ Viral Load (log_10_ Gene Copies/µL)	
	N1	N2	N1	N2	N3	N4	N3	N4
January	30.44	28.44	527.96	71.80	18.59	5910.49	1.27	3.68
February	31.19	33.66	269.35	8.15	1.00	269.35	0.00	2.27
March	34.53	35.20	1.00	8.85	1.00	8.85	0.00	0.95
April	34.53	35.90	81.84	5.71	5.71	86.87	0.76	1.94
May	31.13	31.21	1395.98	141.60	141.60	3503.28	2.15	3.54
June	30.10	32.80	3124.90	79.26	79.26	3124.90	1.90	3.49
July	30.25	27.30	482.64	10.12	8.98	15,405.71	0.95	4.19
August	31.33	28.70	503.10	7.07	2.65	1191.34	0.42	3.08
September	32.39	34.59	428.64	13.81	13.81	1200.21	1.14	3.00
October	25.42	26.41	1.00	65.74	1.00	5476.77	0.00	3.74
November	28.64	35.05	436.34	3.96	1.00	436.34	0.00	2.64
December	35.56	36.88	1.00	1.00	1.00	4.99	0.00	0.65

**Table 3:** COVID-19 testing data from sentinel hospitals in Fuzhou, China, in 2023.

Month	Number of Tests	Number of Positives	Positive Rate (%)
January	167	60	35.93
February	177	16	9.04
March	241	14	5.81
April	206	2	0.97
May	208	90	43.27
June	234	65	27.78
July	193	40	20.73
August	210	55	26.19
September	218	55	25.23
October	219	9	4.11
November	212	0	0.00
December	187	0	0.00

## Data Availability

Data are contained within the article.

## Abbreviations

The following abbreviations are used in this manuscript:

SARS-CoV-2	Severe acute respiratory syndrome coronavirus 2
COVID-19	Coronavirus disease 2019
WBE	Wastewater-based epidemiology
RT-qPCR	Reverse transcription quantitative polymerase chain reaction
N	Nucleocapsid
N3	Lower concentration between N1 and N2 on each sampling day
N4	Higher concentration between N1 and N2 on each sampling day
